# Human *TERT* promoter mutations as a prognostic biomarker in glioma

**DOI:** 10.1007/s00432-021-03536-3

**Published:** 2021-02-06

**Authors:** Branka Powter, Sarah A. Jeffreys, Heena Sareen, Adam Cooper, Daniel Brungs, Joseph Po, Tara Roberts, Eng-Siew Koh, Kieran F. Scott, Mila Sajinovic, Joey Y. Vessey, Paul de Souza, Therese M. Becker

**Affiliations:** 1 Centre for Circulating Tumour Cell Diagnostics and Research, Ingham Institute for Applied Medical Research, 1 Campbell St, Liverpool, NSW 2170 Australia; 2 School of Medicine, Western Sydney University, Campbelltown, NSW 2560 Australia; 3 Western Clinical School, University of New South Wales South, Goulburn St, Liverpool, NSW 2170 Australia; 4 Cancer Therapy Centre, Liverpool Hospital, Elizabeth St and Goulburn St, Liverpool, NSW 2170 Australia; 5 School of Medicine, University of Wollongong, Wollongong, NSW 2522 Australia

**Keywords:** Glioma, Biomarker, TERT promoter mutation, Liquid biopsy, ctDNA

## Abstract

The TERT promoter (*pTERT*) mutations, C228T and C250T, play a significant role in malignant transformation by telomerase activation, oncogenesis and immortalisation of cells. C228T and C250T are emerging as important biomarkers in many cancers including glioblastoma multiforme (GBM), where the prevalence of these mutations is as high as 80%. Additionally, the rs2853669 single nucleotide polymorphism (SNP) may cooperate with these *pTERT* mutations in modulating progression and overall survival in GBM. Using liquid biopsies, *pTERT* mutations, C228T and C250T, and other clinically relevant biomarkers can be easily detected with high precision and sensitivity, facilitating longitudinal analysis throughout therapy and aid in cancer patient management.

In this review, we explore the potential for *pTERT* mutation analysis, via liquid biopsy, for its potential use in personalised cancer therapy. We evaluate the relationship between *pTERT* mutations and other biomarkers as well as their potential clinical utility in early detection, prognostication, monitoring of cancer progress, with the main focus being on brain cancer.

## Introduction

Telomerase reverse transcriptase (TERT) plays an important role in telomere lengthening and oncogenesis in many human cancers (Moyzis et al. 1988). Two particular mutations in the human TERT promoter (*pTERT*) region, C228T and C250T, are important as they promote the formation of a novel binding site for transcriptional enhancers. This in turn drives increased expression and activity of telomerase, an event considered critical for cell immortalisation, and a hallmark of oncogenesis (Hanahan and Weinberg 2011; Huang et al. 2015).

*pTERT* C228T and C250T have been identified in a range of cancers, including primary brain cancers, and are associated with reduced overall survival (OS), suggesting that they may serve as genomic cancer biomarkers (You et al. 2017). Cancer biomarkers are increasingly used in determining disease diagnosis, monitoring of progression, and determining the best outcome-based therapy for patients. Several studies indicate that the presence of *pTERT* mutations are tightly linked with other biomarkers such as EGFR amplification, IDH wild type (in GBM), 1p19q co-deletion, CDKN2A deletion, chromosome 10q loss and SEL1L, suggesting evolutionary co-selection with *pTERT* mutations (Labussière et al. 2014a; Mellai et al. 2020; Nonoguchi et al. 2013). In contrast, there is no association between *pTERT* mutations and other mutations such as *IDH* and *TP53* (Labussière et al. 2014a).

Currently, there are few options for the treatment of brain cancers, regardless of their molecular profile. The Standard-of-care treatment is maximal safe resection (where possible), followed by post-operative radiation and chemotherapy with adjuvant temozolomide (Stupp et al. 2010). Therefore, new treatment approaches are needed, which are tailored for each patient to improve patient outcomes.

In this review, we examine the effects of *pTERT* mutations in various cancers focusing on those originating in brain tissues. We also examine the interaction of *pTERT* mutations with other prognostic biomarkers and their role in cancer progression, OS and potential implementation of *pTERT* mutation screening from liquid biopsies in clinical settings.

## Telomeres and function of telomerase

The telomerase reverse transcriptase (*TERT*) gene, located on chromosome 5p15.33, encodes the catalytic subunit of telomerase, a ribonucleoprotein enzyme essential for the replication of chromosome termini and extension of telomeres in eukaryotic organisms. This function is required for continued cell division and is implicated in cell immortality. Telomeres are chromosome termini that contain repetitive DNA sequences (TTAGGG). The repetitive telomere DNA hexamers occur at chromosomal 3′-ends and can be hundreds and thousands of copies in repetition (Moyzis et al. 1988). Telomeres, without the presence of telomerase function, become progressively shorter during each successive cell division. Loss of telomere length beyond a certain point may cause chromosomal instability and genomic rearrangement. Thus, telomere shortening has important implications for cell proliferation. Once telomere length has reached a critical size (the Hayflick limit) through serial cell divisions, normal cells will undergo irreversible cell cycle arrest, referred to as senescence (Becker and Haferkamp 2013; Hayflick 1965). Senescence prevents further cell proliferation and DNA replication, protecting cells from genetic mutations and chromosome rearrangements which could result in oncogenesis. Telomerase counters telomere loss by stabilising and elongating telomeres through the addition of the telomere repeat of TTAGGG to the 3′ ends of human chromosomes (Blackburn 2005).

Telomerase is turned off in most normal adult human cells due to the transcriptional repression of the *TERT* promoter and are only consistently active in proliferative cells such as germline tissue (ovary and testis), lymphoid lineage-committed progenitor cells, at a low level in normal peripheral leukocytes (including lymphocytes) and bone marrow (Broccoli et al. 1995; Kim et al. 1994; Weng et al. 1996). Reactivation of telomere maintenance mechanisms via telomerase expression is essential for the transformation of normal cells into cancer cells allowing for unlimited cell division and immortality (Counter et al. 1992). In approximately 90% of cancers immortality is achieved by reactivation of telomerase, involving reactivation of the *TERT* gene expression. The remaining cancers use an alternate telomere lengthening (ALT) pathway where homologous recombination occurs to maintain the telomere length (Cesare and Reddel 2010; Heaphy et al. 2011; Patel et al. 2016; Shay and Bacchetti 1997). Reactivation of telomerase reverse transcriptase via *pTERT* alterations plays a pivotal role in gliomas with *pTERT* mutations found in 80–90%, correlating with higher TERT mRNA and protein expression, and subsequent increased telomerase activity. (Borah et al. 2015; Huang et al. 2015; Killela et al. 2013).

## Telomerase promoter mutations

**Fig. 1:**
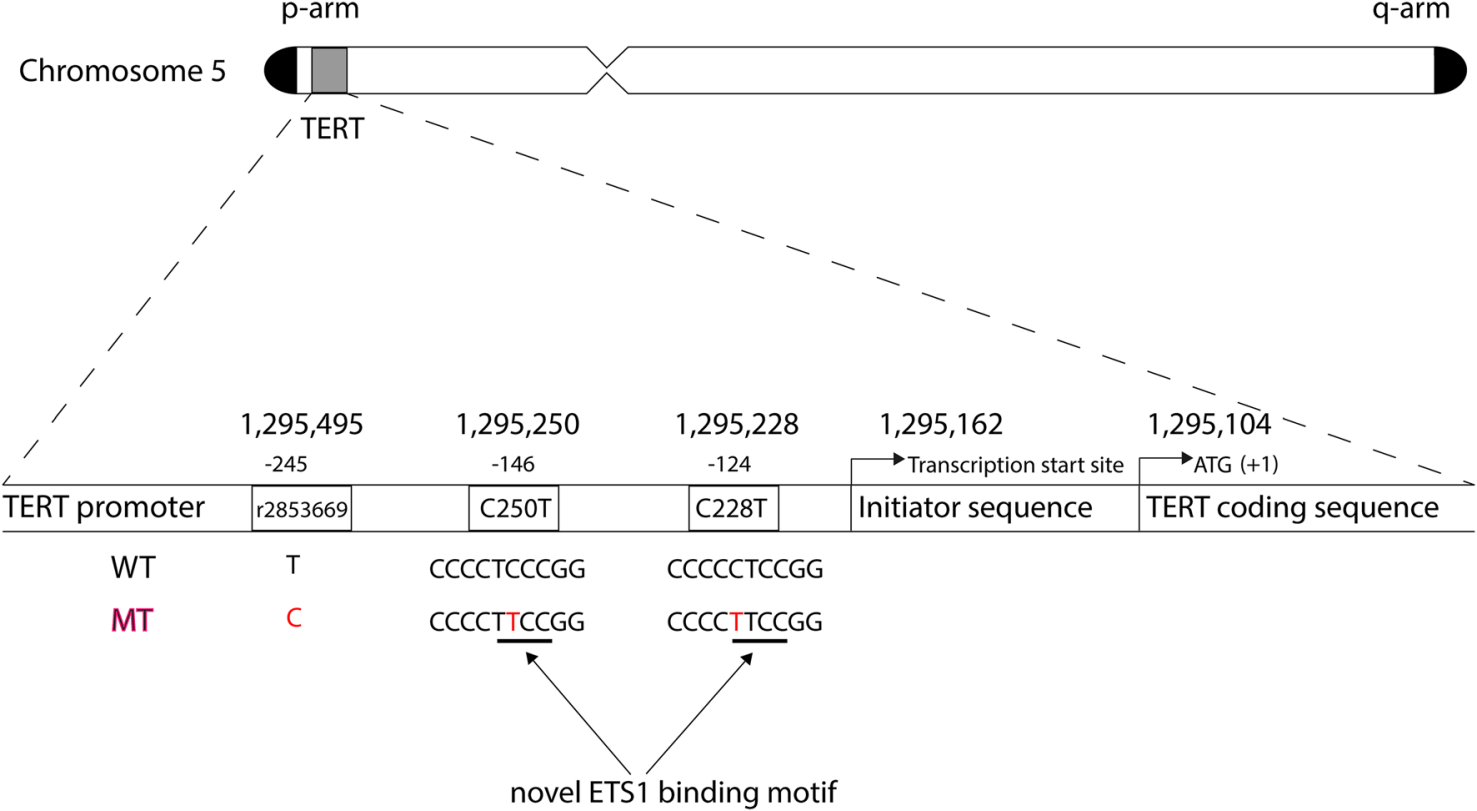
TERT chromosomal location and regulative motives. Schematic illustration showing the TERT promoter with the novel ETS1 binding sites and cancer-specific TERT promoter mutations C250T and C228T. -146 and -124 indicate the position of the C250T and C228T mutations upstream, respectively, in relation to the start of the TERT coding sequence ATG, indicated as + 1. *WT *wild-type *MT *mutation

The *TERT* gene is located on the short arm of chromosome 5 and consists of 16 exons and 15 introns (Cong et al. 1999). The *pTERT*, embedded in a CpG island, located from  – 1800 to + 2300 relative to the ATG start codon, is rich in binding motifs for various transcription factors (Cong et al. 1999) (Fig. 1). In normal human cells p*TERT* is unmethylated at the DNA level, while almost all cancer cells harbour methylated promoter regions. While promoter methylation commonly represses transcription, it results in upregulation of *TERT* expression, possibly by preventing binding of transcriptional repressors to *pTERT* (Lee et al. 2020).

Several studies have identified two specific promoter point mutations (cytosine to thymine substitution), chr5:1,295,228 C > T and chr5:1,295,250 C > T (also denoted C228T and C250T) in cancer cells implicated in the activation of telomerase (Horn et al. 2013; Huang et al. 2013). These two mutations are mutually exclusive. Either mutation increases *TERT* expression, and consequently telomerase activity, and are thought to contribute to tumourigenesis by overcoming cellular senescence and inducing cell immortalisation (Brennan et al. 2013; Huang et al. 2013, 2015). At the molecular level, both *pTERT* mutations create an identical 11 base pair sequence (CCCGGAAGGGG). This constitutes a de novo binding site (Fig. 1) for members of the E26 transformation specific (ETS) family of transcription factors such as the GA-binding protein transcription factor (GABPA), likely involved in transcriptional activation of *TERT* (Bell et al. 2015; Xiao et al. 2002, 2003). GABPA is central to TERT expression in glioblastoma as it had been shown that the knockdown of GABPA significantly reduced mutant promoter activity without affecting wild-type promoter activity (Bell et al. 2015). Furthermore, a tetramer-forming β1L isoform of GABPA is required for full activation of the mutant *pTERT,* while GABPA β1L does not act on the wild-type *pTERT* to induce TERT expression in cell culture experiments (Mancini et al. 2018). Additionally, a recent study found that in the *BRAF*^*V600E*^ mutated glioma cells also carrying *pTERT* mutation, several members of ETS family were hyperactivated: *ETS1*, *GABPA*, *GABPB*, *ETV1*, *ETV4* and *ETV5* (Gabler et al. 2019).

## *pTERT* rs2853669 single nucleotide polymorphism (SNP)

The rs2853669 SNP of *pTERT* is located within a pre-existing ETS2 binding site 5′ to the start codon and close to the C228T and C250T loci -245 bases 5′ to the transcriptional start ATG codon causing a T > C substitution (Fig. 1).

The rs2853669 variant SNP disrupts the endogenous ETS2 transcriptional site, repressing transcriptional activation of *TERT* (Hsu et al. 2006; Nencha et al. 2016). The telomerase activity was observed to be lower in the double C/C homozygous variant of rs2853669 than that of wild type T/T homozygotes indicating disruption of ETS2 binding site and reduced expression of *TERT* (Hsu et al. 2006).

rs2853669 is associated with poorer prognosis and OS, despite lack of association with risk of developing GBM (Mosrati et al. 2015; Spiegl-Kreinecker et al. 2015). This may be due to the rs2853669 SNP modulating negative effects of other oncogenic driver mutation pathways (Mosrati et al. 2015; Spiegl-Kreinecker et al. 2015). A study of 126 GBM patients, showed that the subgroup of patients with wild-type TERT promoter who were also carriers for rs2853669 had longer median survival than those who were non-carriers for rs2853669 (43.5 vs 20.4 months) (Spiegl-Kreinecker et al. 2015). However, when the homozygous CC rs2853669 SNP variant was found survival was significantly shorter, particularly if coinciding with either the C228T or the C250T *pTERT* mutation, which correlated with a very short overall survival median of 8.1 months (Spiegl-Kreinecker et al. 2015). These findings were corroborated by another study showing that patients with homozygous CC rs2853669 SNP variant in the presence of *pTERT* mutation had a similar short overall survival median of 8.2 months (Mosrati et al. 2015). Together these studies imply that the homozygous CC rs2853669 genotype acts as an independent predictor of short patient survival in *pTERT* mutated patients.

Interestingly, data from other studies are conflicting, regarding the survival impact of homozygous, wild type TT rs2853669 SNP with or without *pTERT* mutation (Batista et al. 2016; Simon et al. 2014) (Nencha et al. 2016) indicating further research is required to clarify the impact of this SNP on GBM patient survival.

## *pTERT* mutations in cancer

While *pTERT* mutations are absent in normal human cells (Kim et al. 1994), they are common in many cancers including in glioblastoma (Kim et al. 1994; Liu et al. 2013) (Table 1). *pTERT* mutations were significantly associated with the higher mean age at diagnosis in brain cancers (Vinagre et al. 2013). The C228T mutation is the more prevalent cancer-associated *pTERT*-variant (see Table 1) (Huang et al. 2013; Johanns et al. 2016; Palsgrove et al. 2019). When 887 gliomas were analysed for *pTERT* mutations, C250T was found in 9.5% and C228T in 30.9% of all gliomas, with oligodendrogliomas having the highest proportion of mutations (both mutations combined) of 75.7% (You et al. 2017). *pTERT* C228T was associated with poorer OS compared to patients with C250T gliomas. Furthermore, the study indicated that the *pTERT* mutation frequency increased with age, and younger patients with *pTERT* mutation had longer OS than older patients with *pTERT* mutation (Akyerli et al. 2018). Another study examined 128 GBM samples and detected that 86% had *pTERT* mutations, 75% the C228T and 25% the C250T variant. In this cohort, GBM patients with *pTERT* mutations had shorter OS compared to wild-type *pTERT* patients with median OS 11 versus 20 months, respectively (Mosrati et al. 2015). Other studies confirm this key prognostic role of *pTERT* mutational status in GBM. (Table 1). Interestingly, an in vitro study has shown that programmable base editing of mutated *pTERT* blocked the binding of members of the ETS1 transcription factors to the *TERT* promoter, reduced *TERT* transcription and TERT protein expression, and induced senescence in glioma cell lines, suggesting that targeting *pTERT* mutations could be used as a therapeutic approach in cancer management (Li et al. 2020).

**Table 1:** Prevalence of *pTERT* mutations and their role in oncogenesis and patient outcomes

Cancer	Sample	Number of patients with C228T (%)	Number of patients with C250T (%)	Number of patients with C228T and/or C250T (%)	Outcome/conclusion
Brain – Glioma	Tissue	N/A	N/A	93/199 (46.7%)	Radiomics may be used to predict some molecular subtypes, including the *pTERT* mutation positive and IDH1/2 mutation subtype, with currently limited accuracy. (Arita et al. 2018)
Brain – Glioma	Tissue	32/67 (47.8%)	6/67 (9.0%)	All 38/67 (56.7%)	Patients with *pTERT* mutation demonstrated significantly reduced OS and PFS (median 15 months and 5 months) as compared with those in *pTERT* wild type patients (median 33 months and 31 months) (log rank test: P = 0.031, and P = 0.008, respectively) In grade II tumours, MGMT-unmethylated/*pTERT*-mutated was strongly associated with worse prognosis. (Kim et al. 2018)
				*Grade II 3/9 (33.3%)*	
				*Grade III 8/16 (50%)*	
				*Grade IV 27/42 (64.3%)*	
Brain – Glioma	Tissue	24/56 (42.9%)	10/56 (17.9%)	34/56 (60.7%)	*pTERT* mutations are more common in tumours with high SEL1L expression, which is associated with unfavourable prognosis and worse response to combined radiotherapy and adjuvant temozolomide chemotherapy (Mellai et al. 2020)
Brain – Glioma	Tissue	59/92 (64.1%)	20/92 (21.7%)	72/92 (85.9%)	C228T and C250T were significantly associated with shorter survival in univariate analysis (median 11 vs. 20 months *p* = 0.002 and 12 vs. 20 months, *p* = 0.04 for C228T and C250T, respectively) compared to wild type tumours (Mosrati et al. 2015)
Brain – Glioma All	Tissue	274/887 (30.9%)	84/887 (9.5%)		*pTERT* mutations were detected at a low frequency in Astrocytomas and high in Oligodendrogliomas. *pTERT* mutations were inversely correlated with IDH1/2 mutation. Patients with C250T *pTERT* mutations tended to have longer survival that those with the C225T mutation. The rate of C250T mutations in newly diagnosed gliomas was twice that of recurrent gliomas (You et al. 2017)
*Anaplastic astrocytomas*		*N/A*	*N/A*	*12/37 (32.4%)*	
*Anaplastic oligoastrocytomas*		*N/A*	*N/A*	*34/83 (41%)*	
*Anaplastic oligodendrogliomas*		*N/A*	*N/A*	*10/19 (52.63%)*	
*Oligoastrocytomas*		*N/A*	*N/A*	*112/225 (54.2%)*	
*Oligodendrogliomas*		*N/A*	*N/A*	*53/70 (75.7%)*	
*Primary GBMs*		*N/A*	*N/A*	*89/199 (44.7%)*	
*Secondary GBMs*		*N/A*	*N/A*	*15/51 (29.4%)*	
Brain—Glioblastoma	Tissue	48/74 (64.86)	14/74 (18.92)	62/74 (83.78)	Plays a role in tumourigenesis and pathogenesis of glioblastoma (Liu et al. 2013)
Brain—Glioblastoma	Tissue	17/43 (39.5%)	5/43 (11.6%)	22/43 (51.2%)	TERT mRNA expression higher in patients with *pTERT* mutations C228T (P < 0.0001) C250T (P = 0.0004) (Huang et al. 2015)
Brain All	Tissue	N/A	N/A	68/124 (54.8%)	*pTERT* mutations correlated with telomerase activation and common in glioblastoma, oligodendroglioma, and medulloblastoma (Huang et al. 2015)
*Glioblastoma*		N/A	N/A	*47/56 (83.9%)*	
*Oligodendroglioma*		N/A	N/A	*7/10 (70%)*	
*Diffuse Astrocytoma*		N/A	N/A	*8/40 (20%)*	
*Anaplastic Astrocytoma*		N/A	N/A	*4/12 (33.3%)*	
*Medulloblastoma*		N/A	N/A	*2/6 (33.3%)*	
Brain All		101/166 (60.8%)	20/166 (12.0%)	124/168 (73.8%)	*pTERT* mutations were associated with poorer OS in glioblastoma (*P* = 0.003) and anaplastic astrocytoma (*P* = 0.001), but not in oligodendroglioma (Lee et al. 2017)
*Glioblastoma*		*43/65 (66.2%)*	*11/65 (16.9%)*	*57/65 (87.7%)*	
*Oligodendroglioma*		*55/63 (87.3%)*	*8/63 (12.7%)*	*63/65 (96.9%)*	
*Anaplastic Astrocytoma*		*3/38 (7.9%)*	*1/38 (2.6%)*	*4/38 (10.5%)*	

*N/A*not available, *cfDNA *cell-free DNA, *PFS *progression-free survival

## *pTERT* mutations and association with other prognostic biomarkers

Table 2 summarizes the correlation of *pTERT* mutations and other key biomarkers in gliomas. One key study examined 299 patients with diffuse gliomas and defined them into four distinct molecular groups: *IDH* mutation only (33.8%), *pTERT* mutation only (31.4%), *IDH-pTERT* double mutant (21.4%) or both wild type (13.4%) (Akyerli et al. 2018). Isocitrate Dehydrogenase (*IDH*) mutations are a well-described favourable prognostic marker in glioma (Vuong et al. 2017). Patients with the *IDH-pTERT* double mutations had better overall survival than those with *IDH* only mutations (Akyerli et al. 2018). Moreover, 96.3% of patients with the *IDH-pTERT* double mutations were also positive for 1p/19q co-deletions. All patients that had 1p/19q co-deletions also harboured *pTERT* mutations. More importantly, the analysis showed that the *pTERT* only mutations group was associated with older age and poor OS (Akyerli et al. 2018). These results are supported by the findings of Heidenreich et al*.,* who showed in their cohort of 303 gliomas that the patients with only *pTERT* mutations had worse OS, while the patients with both IDH and *pTERT* mutations had the best OS (Heidenreich et al. 2015). Interestingly, one study showed that combined analysis of IDH1/2 and *pTERT* mutational status could be used to distinguish if a glial lesion is glioma or reactive glioma. The study reported that reactive gliosis samples did not contain C228T or C250T mutations in the TERT promoter region, while 78% of IDH wild type gliomas were found to have *pTERT* mutation (Hewer et al. 2020).

**Table 2:** *pTERT* in glioma and co-occurrence with other biomarkers

Biomarker	Clinical utility	Frequency of co-occurrence	Outcome
IDH1/2	Prognostic marker	Low-grade gliomas had the highest frequency (87.3%) GBM have low frequency (11.5%) of co-occurrence of pTERT mutation with IDH1/2	*IDH*-mut + *pTERT*-mut = better OS, *IDH*-wt + *pTERT*-mut = poorer OS in WHO grade II and IV (Akyerli et al. 2018; Eckel-Passow et al. 2015; Labussière et al. 2014b; Lee et al. 2017; You et al. 2017)
EGFR	Prognostic marker	pTERT-mut and IDH-wt tumours are highly associated with EGFR amplification (44.1%) EGFRvIII is expressed in 24–67% of GBM	*pTERT*-mut + *EGFR*amp = poor OS (Heimberger et al. 2005a,2005b; Labussière et al. 2014b; Pelloski et al. 2007; Wikstrand et al. 1997; You et al. 2017)
PTEN	N/D	54.6% of low-grade gliomas, 71.4% GBM, and all anaplastic gliomas had TERT and PTEN co-mutation	N/D (You et al. 2017)
TP53	N/D	TERT and TP53 mutations in low-grade gliomas and anaplastic gliomas were mutually exclusive, where only 2% and 3.6% of TERT mutation tumours harbored TP53 mutations, respectively	*TP53*-mut + *pTERT*-mut = poor prognosis (You et al. 2017)
ATRX		ATRX mutations and pTERT mutations are mutually exclusive in adult gliomas	N/D (Arita et al. 2013; Killela et al. 2013)
MGMT promoter methylation	Prognostic marker	TERT mutation and MGMT promoter hypermethylation is 51% (25/49) in low-grade glioma and 43.6% (31/71) in GBM	*MGMT* unmethylated/ + *pTERT*-mut = poor prognosis/ worst OS in grade II gliomas (You et al. 2017)
1p19q co-deletion	Prognostic marker	TERT mutation was clustered in tumours with 1p19q LOH (59/99 low-grade gliomas and 8/12 anaplastic gliomas) In 1p/19q-codeleted cases (*n* = 51 [48 oligodendrogliomas and 3 oligoastrocytomas]), the incidence of pTERT-mut was 100% pTERT-mut was identifiable in 87.9% (94 out of 107 of gliomas with 1p19q co-deletion)	1p19qLOH + *pTERT*-mut = better OS (You et al. 2017) (Akyerli et al. 2018) (Labussière et al. 2014b)
SEL1L	Prognostic marker	pTERT mutations associated with SEL1L overexpression	SEL1L overexpression results in tumour progression, cell proliferation and decreased OS and response to therapy (Mellai et al. 2020)

*N/D *not determined, *wt *wild-type, *mut *mutation, *OS *overall survival

A study by You and colleagues analysed the rates and clinical outcomes of combined alterations of pTERT mutations and other key markers including *IDH1/2*, *EGFR*, *TP53*, *PTEN*, *MGMT* and 1p19q, in gliomas. The prognostic impact of *pTERT* mutations varied between groups, with improved prognosis in patients with both *pTERT* and *IDH* mutations, but the poorest survival in patients with *pTERT* mutation and *EGFR* amplification (You et al. 2017).

In contrast, in ALT positive astrocytomas *pTERT* and *IDH1* mutations appeared mutually exclusive and *pTERT* mutations were generally associated with *IDH1* wild-type astrocytoma (Ferreira et al. 2020). This study indicates that ALT may be the major telomere maintenance mechanism in *IDH1* mutation astrocytoma resulting in histidine substitution at arginine 132 (IDH1^R132H^) mutated astrocytomas and that IDH1^R132H^ downregulates ATRX expression in vitro resulting in ALT (Ferreira et al. 2020). This potentially contributes to the association of IDH1^R132H^ mutations, α-thalassemia/mental retardation syndrome X-linked (ATRX) loss and ALT (Ferreira et al. 2020). The *ATRX* gene is frequently mutated in gliomas and while its role in gliomagenesis is not clear so far, it is thought to be associated with ALT (Koschmann et al. 2016; Rizzo et al. 2009). A report into the prevalence of ALT mechanism in human cancers found 11% of adult glioblastoma rely on ALT, while the majority of adult glioblastoma relies on the reactivation of telomerase (Heaphy et al. 2011).

As mentioned above, there is a correlation between *pTERT* and SNP variant rs2853669, with SNP alone associated with improved OS in glioma, while *pTERT* decreases OS and *pTERT* together with wild-type SNP rs2853669 further lowers OS in glioma (Rachakonda et al. 2013).

*Suppressor of Lin‐12‐like protein (C. elegans)* (SEL1L) is recently emerging as a potential biomarker in brain cancer. SEL1L expression is associated with glioma proliferation and severity, as seen in the human brain glioblastoma cells cultured in vitro and in a formalin-fixed paraffin sections of glial tumours (Cattaneo et al. 2014). A recent study has shown that *pTERT* mutations are associated with SEL1L overexpression in glioblastoma; high SEL1L immunoreactivity correlates with tumour progression, cell proliferation, decreased OS and poorer response to therapy (Mellai et al. 2020). Further, it was proposed that SEL1L could be an important biomarker in *pTERT* mutant/*EGFR* amplified/*IDH* wild-type subgroup of glioblastoma (Mellai et al. 2020).

## Detection of *TERT* promoter mutations in liquid biopsies

Currently brain cancer is diagnosed via magnetic resonance imaging (MRI) and, or computerised tomography (CT). A definite diagnosis of glioblastoma is obtained by histopathological confirmation at surgery or biopsy. There is an expanding role for molecular biomarker tests to aid treatment decisions and prognostication such as *IDH1/2* mutation, *MGMT promoter* methylation, 1p19q co-deletion, *pTERT* (C228T, C250T), *H3.3* (K27M and G34R/V), as well as Next Generation Sequencing Glioma Panel using these tumour tissue samples. Improved biomarker testing is of increasing interest in clinical trials worldwide in glioblastoma (such as Visual Study of Molecular Genotype in Glioma Evolution, NCT03750890) and other cancers (Visual Study of Molecular Genotype in Glioma Evolution).

However, tissue biopsies may poorly reflect tumour heterogeneity. Further, these biopsies are invasive and cancer patient condition or tumour location may be risky and/or prohibitive.

Liquid biopsy is an alternative way of examining molecular tumour profiles and utilises blood, cerebrospinal fluid (CSF), urine and other bodily fluids for detection and isolation of circulating tumour cells (CTCs) and circulating tumour DNA (ctDNA) from cancer patients. ctDNA release into blood and bodily fluids depend on the location of the tumour, size and the vascular infiltration of the tumour (Haber and Velculescu 2014). Analysis of ctDNA in real time can provide important molecular insights into the tumour composition, heterogeneity, prognostic biomarkers and their association with other clinically relevant cancer biomarkers. Previous studies have shown that the levels of ctDNA present in liquid biopsies vary from patient to patient, however, the relative levels on repeated sampling in a single patient can indicate cancer progression (Diehl et al. 2008). Monitoring tumour dynamics via ctDNA before, during and post-treatment serves as an important tool. Using sensitive techniques such as droplet digital PCR has made this much easier as it can detect minute amounts of ctDNA in liquid biopsies (Ding et al. 2018). Accordingly, the potential of screening for *pTERT* mutations as biomarkers for future individualised therapies for patients should be considered. Increasing technological development, in the area of liquid biopsies, allow for a more convenient method of biomarker detection and are increasingly being adopted in clinical trials worldwide.

In brain cancer, liquid biopsy analysis may be more challenging due to the blood–brain barrier preventing release of tumour derived entities into the blood. Nevertheless, we and others have shown that CTCs and ctDNA can be isolated and analysed from brain cancer patients (Lynch 2020; Macarthur et al. 2014; Müller et al. 2014; Sareen et al. 2020; Sullivan et al. 2014). Further, ctDNA detection could predict the recurrence of disease earlier than conventional methods of monitoring in many cancer types (Ding et al. 2019; Gao et al. 2016; McEvoy et al. 2019; Sozzi et al. 2001; Tie et al. 2016; Wang et al. 2015). A study analysing the overall detection rate of ctDNA in the 419 primary brain tumours, including 222 glioblastomas, have shown that the detection of genomic alteration via ctDNA is achievable, with 211 patients showing some genomic alteration (Piccioni et al. 2019). Another study, looking at the clinical utility of plasma cell-free DNA (cfDNA) in adult patients with newly diagnosed glioblastoma, have determined that the patients had higher plasma cfDNA concentration at baseline (Bagley et al. 2020). The high baseline plasma cfDNA is associated with the worse progression-free survival with a median of 4.9 months vs 9.5 months, inducating that the plasma taken at that point may be an informative prognostic tool (Bagley et al. 2020). Furthermore, a recent study on diffuse gliomas, indicate that the presence of the ctDNA in CSF may serve as an early indicator of progression in glioma (Miller et al. 2019).

Thus, liquid biopsy may play a major role in diagnosis, monitoring, assessing disease progression and predicting response to brain cancer treatments in the future. Recently, ctDNA isolated from plasma has been successfully screened for *pTERT* mutations in various cancers, including metastatic melanoma, hepatocellular carcinoma, myxoid liposarcomas and urothelial cancer (Barata et al. 2017; Braig et al. 2019; Calapre et al. 2019; Ikeda et al. 2018; McEvoy et al. 2017). *pTERT* mutations can be detected using digital droplet PCR assays implying its potential utility in brain cancer therapy decision making and in progression monitoring (Braig et al. 2019; Calapre et al. 2019; Deniel et al. 2019; Hayashi et al. 2019; Wan et al. 2017).

## Conclusion

*pTERT* mutations, C228T and C250T, frequently occur in many cancers, including brain cancers such as glioblastoma (Arita et al. 2013; Kim et al. 1994; Panebianco et al. 2019; Vinagre et al. 2013). These mutations induce the novel ETS1 binding site, which increases the expression of telomerase directly contributing to tumorigenesis, and are associated with poorer OS (Bell et al. 2015; Huang et al. 2013). Presence of *pTERT* mutations correlates with the presence of other biomarkers, such as *IDH1*, 1p19q, *TP53*, *EGFR.* Screening for the presence of variants in all of these genes may help prognosticate patients which may, in turn, improve clinical decision making (Arita et al. 2018, 2016; Heidenreich et al. 2015; Hewer et al. 2020; Kim et al. 2018; Mosrati et al. 2015; Pelloski et al. 2007; Spiegl-Kreinecker et al. 2015; Yuan et al. 2016). Whilst utility of liquid biopsies, as a minimally invasive approach, in the brain cancer setting is in its infancy, CTC and ctDNA analyses in brain cancer are increasingly common (Sareen et al. 2020). This review shows the detection of ctDNA and cfDNA in plasma and CSF of glioma patients is possible, and hand in hand with improved molecular detection techniques may become an important tool in determining prognosis and progression-free survival (Bagley et al. 2020; Miller et al. 2019).

The investigation of the profile of various biomarkers may hold clues to better understand tumour biology and may predict benefit of potential combination therapies. This area should therefore be a focus of further studies.
